# Efficacy and safety of immune checkpoint inhibitors combined with chemotherapy versus bevacizumab combined with chemotherapy in first-line treatment of driver gene-negative advanced non-squamous non-small cell lung cancer: a single-center retrospective study

**DOI:** 10.3389/fonc.2026.1898071

**Published:** 2026-08-13

**Authors:** Wenqiang Mo, Yinzhen Li, Luna Zhao, Yuan Liu, Qi Shu, Jing Fei

**Affiliations:** The First Affiliated Hospital of Shihezi University, Shihezi, China

**Keywords:** bevacizumab, efficacy, immune checkpoint inhibitors, non-squamous non-small cell lung cancer, safety

## Abstract

**Purpose:**

To compare the efficacy and safety of immune checkpoint inhibitors (ICIs) versus bevacizumab (both combined with chemotherapy) for first-line treatment of driver gene-negative advanced non-squamous non-small cell lung cancer (NSCLC).

**Methods:**

This single-center retrospective study enrolled 194 patients with driver gene-negative advanced non-squamous NSCLC treated at our hospital from May 1, 2019, to May 1, 2025: 79 received ICIs plus chemotherapy, and 115 received bevacizumab plus chemotherapy. Both groups received 4–6 cycles of combination therapy followed by maintenance therapy until disease progression or the end of the study. The primary endpoint was progression-free survival (PFS), and secondary endpoints included objective response rate (ORR), disease control rate (DCR), and adverse events (AEs).

**Results:**

Short-term efficacy: ORR was significantly higher in the ICIs plus chemotherapy group than in the bevacizumab plus chemotherapy group (41.77% vs 26.09%, *P* = 0.022), while DCR showed no statistically significant difference between groups (84.81% vs 79.13%, *P* = 0.317). Survival benefit: Median PFS was 14.330 months in the ICIs plus chemotherapy group, which was superior to 9.60 months in the bevacizumab plus chemotherapy group (*P* = 0.014). Safety: The ICIs plus chemotherapy group had a higher incidence of thyroid dysfunction (8.86% vs 0%, *P* = 0.002), immune-related pneumonitis (3.80% vs 0%, *P* = 0.066) and immune-related hepatitis (5.06% vs 0%, *P* = 0.026); the bevacizumab plus chemotherapy group had a higher incidence of proteinuria (7.83% vs 0%, *P* = 0.012) and hypertension (6.96% vs 0%, *P* = 0.022). Other AEs were comparable between the two groups. Subgroup analysis: Higher disease progression risk was observed in the bevacizumab plus chemotherapy group for patients with PD-L1 high expression (HR = 3.731, *P* = 0.039), indicating this population benefits more from ICIs plus chemotherapy. Higher progression risk was seen in the ICIs plus chemotherapy group for males (HR = 2.410, *P* = 0.015), ever-smokers (HR = 2.215, *P* = 0.047), patients with ECOG score 0-1 (HR = 1.762, *P* = 0.032) and patients without bone metastasis (HR = 2.112, *P* = 0.036), suggesting these subgroups are more likely to benefit from bevacizumab plus chemotherapy.

**Conclusions:**

For patients with driver gene-negative advanced non-squamous NSCLC, ICIs combined with chemotherapy are superior to bevacizumab combined with chemotherapy in terms of short-term efficacy and progression-free survival, with manageable but distinct adverse event profiles.

## 1 Introduction

Lung cancer is one of the most burdensome malignant tumors worldwide, of which non-small cell lung cancer (NSCLC) is the most common histological type. Its high incidence and mortality continue to place significant pressure on public health systems and socioeconomic development (1). NSCLC is histologically divided into two major subtypes: squamous cell carcinoma and non-squamous cell carcinoma. Non-squamous NSCLC mainly includes adenocarcinoma, large cell carcinoma, and other less common subtypes, with adenocarcinoma being the most prevalent. Although systemic treatment for advanced NSCLC has evolved continuously over the past decade, the overall prognosis remains poor. Clinical practice thus needs to explore more effective and widely applicable first-line strategies (2). For driver gene-negative advanced non-squamous NSCLC, platinum-based doublet chemotherapy has long been the standard first-line regimen, but this strategy provides limited survival benefits and its toxicities cannot be ignored. This reality constitutes an important background for the continuous development of new treatment models (3).

In recent years, immune checkpoint inhibitors (ICIs), especially antibody drugs targeting programmed cell death protein-1 (PD-1) or programmed death-ligand 1 (PD-L1), have become an important pathway for first-line treatment of driver gene-negative advanced non-squamous NSCLC. Multiple prospective randomized controlled trials (RCTs) have shown that compared with chemotherapy alone, immune checkpoint inhibitors combined with chemotherapy can improve patients’ progression-free survival (PFS) and overall survival (OS), as well as increase objective response rate (ORR) (4, 5). Among them, key phase III studies such as KEYNOTE-189 and IMpower130 have further established the clinical status of this regimen in the first-line treatment of driver gene-negative advanced non-squamous NSCLC (6, 7).

In addition to ICIs combined with chemotherapy, bevacizumab combined with chemotherapy represents another key important option for first-line treatment in this population. As an anti-angiogenic drug targeting vascular endothelial growth factor (VEGF), bevacizumab has demonstrated survival benefits when combined with chemotherapy – particularly in non-squamous NSCLC patients (8, 9). Therefore, for driver gene-negative advanced non-squamous NSCLC, both ICIs-chemotherapy and bevacizumab-chemotherapy regimens are clinically established, making comparative efficacy and safety analyses of clear clinical significance.

However, current comparative evidence for these two combination regimens remains insufficient. Previous meta-analyses and network meta-analyses mainly relied on indirect comparisons to evaluate the efficacy and safety of different first-line regimens for driver gene-negative advanced non-squamous NSCLC. Nevertheless, their conclusions are often limited by high inter-study heterogeneity, significant differences in baseline patient characteristics, and restricted data availability—all of which affect evidence robustness and clinical decision-making support. Existing studies, such as Yu et al.’s indirect meta-analysis and Zhai et al.’s Bayesian network meta-analysis, have compared ICIs-chemotherapy versus bevacizumab-chemotherapy; however, their conclusions regarding population extrapolation and discriminatory power in specific subgroups still require verification by higher-quality evidence (10, 11).

Furthermore, existing comparative studies are primarily based on randomized controlled trial (RCT) data, whereas patient populations in real-world research are often more complex. Patients in clinical practice may exhibit more comorbidities, varied functional status, and modified treatment pathways; consequently, their characteristics frequently diverge from RCT inclusion/exclusion criteria. Therefore, the applicability of RCT-derived conclusions in real-world settings remains limited. This gap between the ‘trial environment’ and ‘actual clinical practice’ necessitates direct comparison of the two first-line combination strategies using real-world data.The rationale for comparing these two regimens is further informed by the differences between international and Chinese guideline frameworks. Current NCCN and ESMO guidelines recommend chemo-immunotherapy as the standard first-line treatment for patients without actionable driver alterations, with ICI monotherapy as an option for selected patients with high PD-L1 expression (≥50%), while bevacizumab combined with chemotherapy is generally reserved for patients with contraindications to immunotherapy or in selected clinical situations. In contrast, the Chinese Society of Clinical Oncology (CSCO) NSCLC guidelines (2024 version) list both ICIs combined with chemotherapy and bevacizumab combined with chemotherapy as recommended first-line options (Level I recommendation) for driver gene-negative advanced non-squamous NSCLC. This parallel recommendation reflects several considerations unique to the Chinese clinical context: a substantial proportion of patients present with contraindications to immunotherapy (e.g., pre-existing autoimmune conditions, severe organ dysfunction, or active viral hepatitis), for which bevacizumab-based regimens serve as a necessary alternative; regional disparities in ICI accessibility and reimbursement coverage still influence treatment selection in real-world practice, particularly in western China; the availability of locally developed anti-angiogenic biosimilars has improved the affordability of bevacizumab-based therapy; and the diverse PD-L1 testing landscape and variable expression levels create clinical scenarios in which bevacizumab-chemotherapy may be preferred (e.g., PD-L1-negative tumors). These factors underscore the clinical relevance of directly comparing the two regimens in a real-world Chinese cohort, particularly from western China, where treatment patterns may differ from those in eastern coastal regions.

Based on the above background, this study adopts a retrospective design. It aims to directly compare the efficacy and safety of ICIs combined with chemotherapy versus bevacizumab combined with chemotherapy in patients with driver gene-negative advanced non-squamous NSCLC in real-world frontline treatment settings, and to further explore potential beneficiary populations-providing evidence with greater practical value for the formulation of personalized treatment plans and optimization of clinical decision-making.

## 2 Research subjects and methods

This study used a retrospective cohort study design and included a total of 194 patients with advanced non-squamous NSCLC who received first-line treatment with ICIs combined with chemotherapy or bevacizumab combined with chemotherapy at the First Affiliated Hospital of Shihezi University between May 1, 2019, and May 1, 2025. Baseline characteristics of all study subjects were analyzed (**Table 1**).

**Table 1:** Baseline characteristics of patients in both groups.

Variable	IC group	BC group	χ^2^/F	P
Gender			0.109	0.741
Male	54 (68.35%)	76 (66.09%)		
Female	25 (31.65%)	39 (33.91%)		
Age	65 (54, 76)	64 (59, 69)	-0.555	0.579
Family history of malignant tumors			1.377	0.241
No	74 (93.67%)	102 (88.70%)		
Yes	5 (6.33%)	13(11.30%)		
History of Alcohol Consumption			1.151	0.283
No	55 (69.62%)	88 (76.52%)		
Yes	24 (30.38%)	27 (23.48%)		
Smoking history			1.856	0.173
No	32 (40.51%)	58 (50.43%)		
Yes	47 (59.49%)	57 (49.57%)		
Hypertension			1.477	0.224
No	52 (65.82%)	85 (73.91%)		
Yes	27 (34.18%)	30 (26.09%)		
Heart disease			3.468	0.063
No	70 (88.61%)	90 (78.26%)		
Yes	9 (11.39%)	25 (21.74%)		
Diabetes			0.028	0.867
No	68 (86.08%)	98 (85.22%)		
Yes	11 (13.92%)	17 (14.78%)		
Chronic Obstructive Pulmonary Disease			2.051	0.152
No	66 (83.54%)	104 (90.43%)		
Yes	13 (16.46%)	11 (9.57%)		
Bronchial asthma			4.436	0.066
No	76 (96.20%)	115 (100.00%)		
Yes	3 (3.80%)	0 (0.00%)		
Chronic bronchitis			1.144	0.285
No	72 (91.14%)	99 (86.09%)		
Yes	7 (8.86%)	16 (13.91%)		
Brain metastasis			2.904	0.088
No	71 (89.97%)	93 (80.87%)		
Yes	8 (10.13%)	22 (19.13%)		
Lung metastasis			1.869	0.172
No	65 (82.28%)	85 (73.91%)		
Yes	14 (17.72%)	30 (26.09%)		
Liver metastasis			1.515	0.218
No	72 (91.14%)	98 (85.22%)		
Yes	7 (8.86%)	17 (14.78%)		
Bone metastasis			3.550	0.060
No	59 (74.68%)	71 (61.74%)		
Yes	20 (25.32%)	44 (38.26%)		
Lymphatic metastasis			0.800	0.371
No	53 (67.09%)	84 (73.04%)		
Yes	26 (32.91%)	31 (26.96%)		
Other metastatic sites			0.296	0.586
No	68 (86.08%)	102 (88.70%)		
Yes	11 (13.92%)	13 (11.30%)		
ECOG Score			1.256	0.262
0–1 Point	54 (68.35%)	87 (75.65%)		
≥2 Point	25 (31.65%)	28 (24.35%)		
Pathological differentiation			2.325	0.313
Poorly differentiated	50 (63.29%)	63 (54.78%)		
moderately differentiated	20 (25.32%)	41 (35.65%)		
Highly differentiated	9 (11.39%)	11 (9.57%)		
Pathological type			0.029	0.864
Adenocarcinoma	74 (93.67%)	107 (93.04%)		
Other	5 (6.33%)	8 (6.96%)		

The inclusion criteria for study subjects are as follows: age ≥18 years; confirmed diagnosis of advanced non-squamous NSCLC by histopathology or cytology, staged as stage IV according to the 8th edition TNM staging system of the International Association for the Study of Lung Cancer; genetic testing results confirm the absence of known actionable driver gene mutations, such as EGFR sensitive mutations, ALK rearrangements, and ROS1 rearrangements; no prior systemic anti-tumor treatment for advanced NSCLC; expected survival of at least 3 months; at least one measurable lesion according to the Response Evaluation Criteria in Solid Tumors (RECIST) version 1.1.

The exclusion criteria are as follows: history of other active malignant tumors; presence of active autoimmune diseases or active autoimmune diseases requiring long-term systemic glucocorticoid therapy; presence of active hemoptysis, high risk of severe bleeding, or uncontrolled blood pressure; concomitant severe cardiovascular diseases, severe infections, active tuberculosis, or other underlying conditions unsuitable for the treatment regimen of this study; known contraindications or severe allergy to any of the drugs involved in this study; incomplete clinical medical records or missing follow-up information, making it impossible to effectively evaluate efficacy and safety.

This study has been approved by the Ethics Committee of the First Affiliated Hospital of Shihezi University, with the ethics approval number KJ2025-607-01. The implementation of the study strictly follows the ethical principles established by the Declaration of Helsinki, and all patients signed written informed consent for treatment.

### 2.1 Treatment plan

All patients received 4 to 6 cycles of combination therapy during the induction treatment phase and were assigned to either the ICIs plus chemotherapy group (IC group) or the bevacizumab plus chemotherapy group (BC group). Both groups received platinum-based doublet chemotherapy as the backbone treatment, with specific chemotherapy regimens including paclitaxel combined with cisplatin or carboplatin, and pemetrexed combined with cisplatin or carboplatin.

In the IC group, patients continued to receive ICI maintenance therapy after the completion of induction therapy until clinical benefit was lost. The ICIs used included pembrolizumab, atezolizumab, toripalimab, sintilimab, camrelizumab, and tislelizumab.

In the BC group, patients continued to receive bevacizumab maintenance therapy after completing induction treatment, until the occurrence of uncontrollable toxic reactions or disease progression.

Before the start of each treatment cycle, all patients underwent a systematic evaluation, including complete blood count, liver and kidney function tests, as well as chest CT or PET/CT scans, to assess the condition of the body and tumor changes. After each chemotherapy session, patients were observed in the observation area for 2–4 hours to monitor vital signs, allergic reactions, and post-injection responses, in order to promptly identify and manage.

### 2.2 Evaluation and data collection

#### 2.2.1 Follow-up

All patients attended regular outpatient follow-ups during the treatment period and underwent clinical assessments on Day 1 of each treatment cycle. Imaging reviews were conducted once every 2 treatment cycles, including chest and abdominal CT scans and cranial MRI examinations, continuing until disease progression or study termination. The follow-up cutoff date was December 31, 2025.

#### 2.2.2 Tumor response assessment

The efficacy of tumor treatment was evaluated according to the RECIST 1.1 criteria (12). The efficacy was graded into complete response (CR), partial response (PR), stable disease (SD), and progressive disease (PD).

Objective Response Rate (ORR) is defined as the proportion of all evaluable patients who achieve complete response or partial response. Disease Control Rate (DCR) is defined as the sum of the proportions of complete response, partial response, and stable disease. Progression-Free Survival (PFS) is defined as the time from the start of initial first-line treatment to disease progression or death from any cause.

Adverse events were evaluated according to the National Cancer Institute’s Common Terminology Criteria for Adverse Events version 5.0 (13). The primary endpoints of this study were progression-free survival (PFS), while the secondary endpoints were objective response rate (ORR), disease control rate (DCR) and adverse events (AEs).

#### 2.2.3 Data collection

The data for this study all were sourced from the Hospital Information System(HIS). The collected content mainly included demographic characteristics, clinical features, and pre-treatment examination data.

Demographic characteristics include gender, age, family history of malignant tumors, history of alcohol consumption, and smoking history. Clinical characteristics include underlying conditions such as comorbidities. Baseline assessment data include physical examination results, complete blood counts, serum biochemical indicators, bronchoscopy findings, and imaging examination data, including chest CT, abdominal ultrasound or CT, bone scans, and brain MRI.

#### 2.2.4 Statistical analysis

Statistical analysis was performed using SPSS 27.0 and GraphPad Prism 9.0. Normally distributed continuous variables were expressed as mean ± standard deviation and compared using independent t-tests; non-normally distributed data were expressed as median (interquartile range) and compared using Mann-Whitney U tests. Categorical data were expressed as frequency (percentage) and compared using χ² or Fisher’s exact tests.

For survival analysis, progression-free survival (PFS) curves were generated using the Kaplan-Meier method, with between-group differences assessed by the log-rank test. Cox proportional hazards regression models were subsequently employed to calculate hazard ratios (HRs) and 95% confidence intervals (CIs) for evaluating treatment regimen effects on survival outcomes.

Stratified subgroup analyses evaluated the impact of PD-L1 expression levels on treatment efficacy and safety, categorizing expression status as high expression, positive, negative, or unknown. Additional subgroup analyses assessed clinicopathological predictors including gender, smoking history, Eastern Cooperative Oncology Group (ECOG) performance status, and brain/liver/bone metastasis to identify outcome-influencing factors. Results were visualized in forest plots, with all statistical tests being two-tailed at significance level α=0.05.

## 3 Results

### 3.1 Characteristics of the patient

This study enrolled 194 patients, with 79 assigned to the IC group and 115 to the BC group. The median age was 65 years (range: 54-76) in the IC group and 64 years (range: 59-69) in the BC group. The cohort comprised 130 males (66.3%) and 64 females (33.7%). Baseline characteristics showed no significant differences between groups (all *P*>0.05), confirming comparability (detailed in **Table 1**).

### 3.2 Clinical efficacy

**Figure 1:**
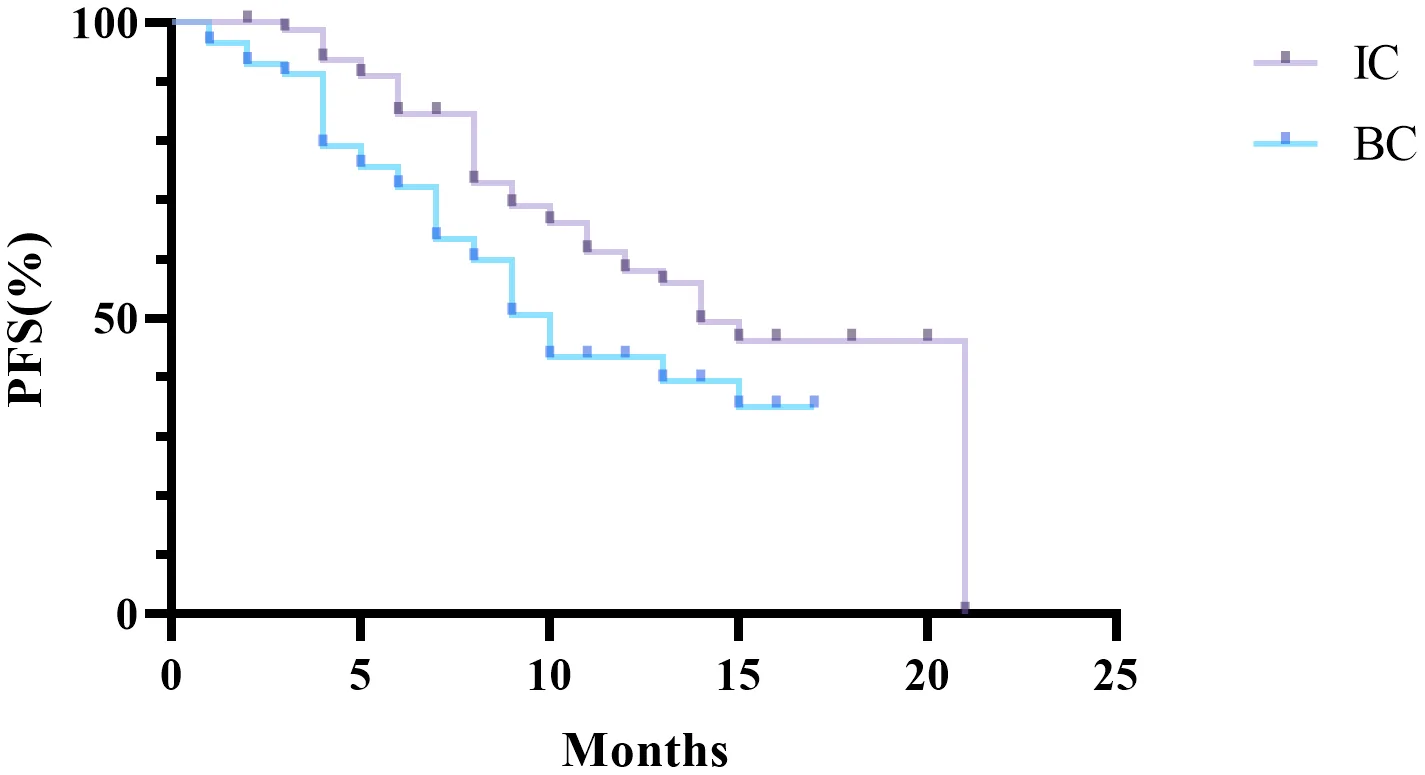
Kaplan-Meier survival curves for progression-free survival (PFS). Kaplan-Meier estimates of PFS comparing the ICIs combined with chemotherapy group (IC group, n=79) with the bevacizumab combined with chemotherapy group (BC group, n=115). The x-axis represents time from initiation of first-line treatment (months); the y-axis represents the probability of progression-free survival. The median PFS was 14.33 months (95% CI: 12.525-16.135) in the IC group and 9.60 months (95% CI: 8.489-10.711) in the BC group. The between-group difference was assessed using the Log-rank test and was statistically significant (P = 0.014). The IC group is represented by the purple line and the BC group by the blue line; short vertical tick marks indicate censored observations. PFS, progression-free survival; IC group, immune checkpoint inhibitors combined with chemotherapy group; BC group, bevacizumab combined with chemotherapy group; CI, confidence interval.

All patients completed follow-up without loss. For the primary efficacy endpoint, median progression-free survival (PFS) was 14.330 months (95% CI: 12.525-16.135) in the IC group versus 9.600 months (95% CI: 8.489-10.711) in the BC group. The intergroup difference was statistically significant (*P* < 0.05), indicating longer median PFS with IC therapy. Detailed results are presented in **Table 2**, and the Kaplan-Meier survival curve for PFS is shown in **Figure 1**.

**Table 2:** Survival analysis of the primary outcome events in both patient groups.

Grouping	mPFS (95% CI)	χ^2^	P	HR	P
IC Group	14.330 (12.525, 16.135)	6.062	0.014	1.653	0.016
BC Group	9.600 (8.489, 10.711)				

**Figure 2:**
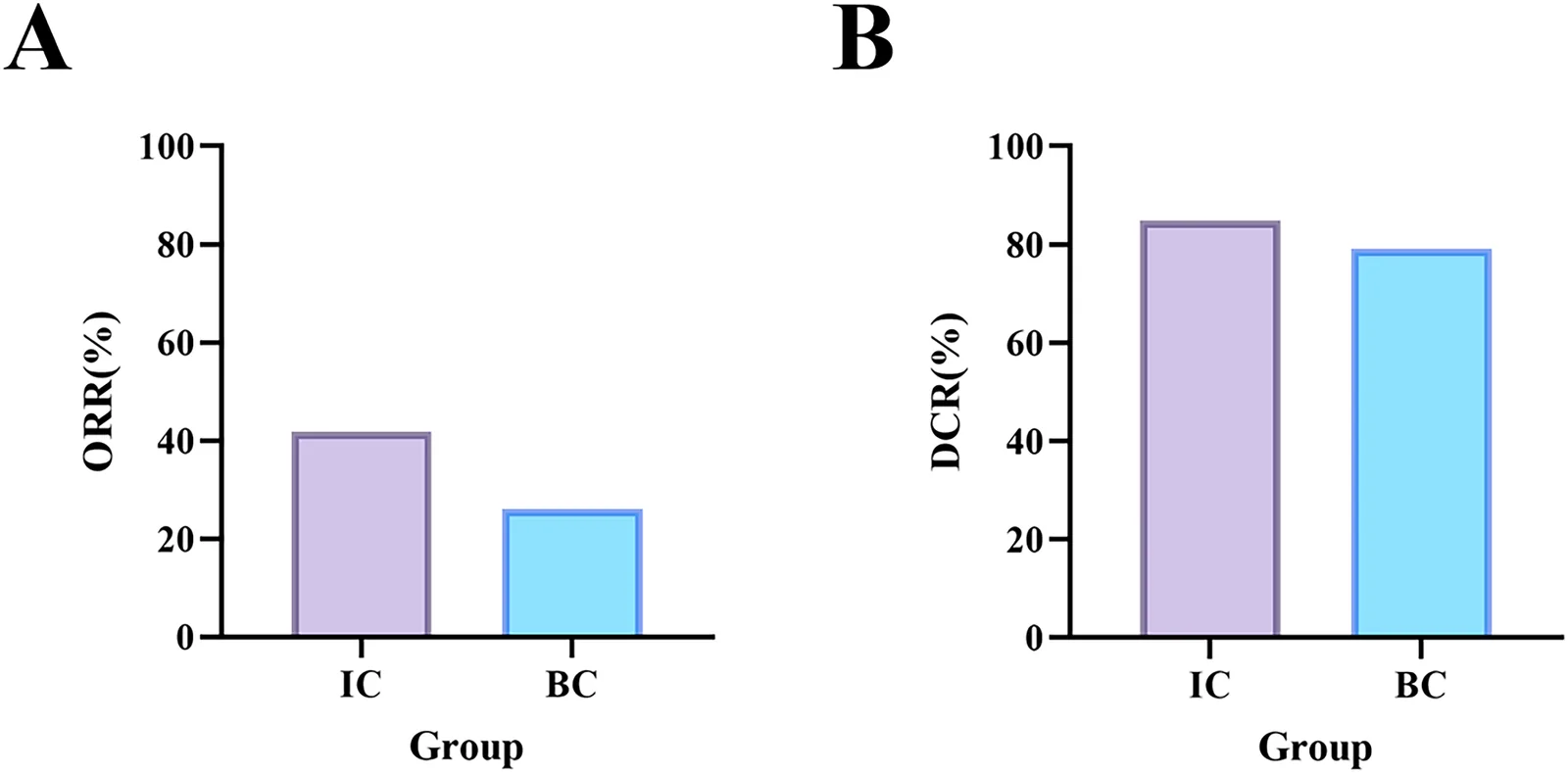
Comparison of short-term efficacy between the two groups. **(A)** Objective response rate (ORR): The ORR was 41.77% in the IC group (n=79) and 26.09% in the BC group (n=115), with a statistically significant between-group difference (chi-square test, P = 0.022). ORR is defined as the proportion of patients achieving complete response (CR) or partial response (PR) among all evaluable patients. **(B)** Disease control rate (DCR): The DCR was 84.81% in the IC group and 79.13% in the BC group, with no statistically significant between-group difference (chi-square test, *P* = 0.317). DCR is defined as the proportion of patients achieving CR, PR, or stable disease (SD). The y-axis of the bar chart represents percentage (%). The IC group is represented by purple bars and the BC group by blue bars. ORR, objective response rate; DCR, disease control rate; IC group, immune checkpoint inhibitors combined with chemotherapy group; BC group, bevacizumab combined with chemotherapy group; CR, complete response; PR, partial response; SD, stable disease.

In terms of short-term response, no patients in the entire cohort achieved CR. In the IC group, there were 33 cases of PR (41.77%), 34 cases of SD (43.04%), and 12 cases of PD (15.19%), with an ORR of 41.77% and a DCR of 84.81%. In the BC group, there were 30 cases of PR (26.09%), 61 cases of SD (53.04%), and 24 cases of PD (20.87%), with an ORR of 26.09% and a DCR of 79.13%. Comparison between the two groups showed that the difference in ORR was statistically significant (*P* = 0.022), while the difference in DCR was not statistically significant (*P* = 0.317). Detailed results are presented in **Table 3**, **Figures 2A, B**.

**Table 3:** Comparison of short-term response between the two patient groups.

Efficacy	IC group	BC group	χ^2^	P
CR	0	0	5.319	0.070
PR	33 (41.77)	30 (26.09)		
SD	34 (43.04)	61 (53.04)		
PD	12 (15.19)	24 (20.87)		
ORR	33 (41.77)	30 (26.09)	5.254	0.022
DCR	67 (84.81)	91 (79.13)	1.000	0.317

Given PD-L1’s established role as an immunotherapy biomarker, we analyzed prognostic differences by PD-L1 expression status (**Table 4**). Stratified analysis revealed that in high PD-L1 expressers, bevacizumab therapy was associated with significantly worse progression-free survival (HR = 3.731; 95% CI: 1.069-13.022; *P* = 0.039), indicating reduced treatment benefit. Conversely, no significant PFS differences were observed in patients with low or negative PD-L1 expression.

**Table 4:** Therapeutic efficacy in patients with varying PD-L1 expression levels.

PD-L1 TPS	B	SE	Wald	P	HR	95% CI	
						Lower limit	Upper limit
Unknown	0.586	0.610	0.924	0.336	1.798	0.544	5.942
Negative	0.446	0.296	2.267	0.132	1.563	0.874	2.794
Positive	0.712	0.476	2.244	0.134	2.039	0.803	5.179
High expression	1.317	0.638	4.263	0.039	3.731	1.069	13.022

### 3.3 Safety assessment

Overall, the incidence of adverse events in the IC group was similar to that in the BC group.

Regarding common chemotherapy-related adverse events, the most frequently observed adverse events in the IC group included neutropenia in 16 cases (20.25%), anemia in 15 cases (18.99%), nausea in 14 cases (17.72%), vomiting in 13 cases (16.46%), and leukopenia in 11 cases (13.92%). The most frequent adverse events in the BC group included leukopenia in 24 cases (20.87%), nausea in 23 cases (20.00%), vomiting in 19 cases (16.54%), neutropenia in 16 cases (13.91%), and anemia in 14 cases (12.17%). Most of the above adverse events were considered related to chemotherapy, so the differences between the two groups were not statistically significant.

Regarding immune-related adverse events, 3 patients (3.80%) in the IC group developed immune-related pneumonitis, 4 patients (5.06%) developed immune-related hepatitis, and 7 patients (8.86%) had thyroid dysfunction; no such events were observed in the BC group. These events were considered immune therapy-specific adverse events, and most of the differences between the two groups were statistically significant, with P-values of 0.066, 0.026, and 0.002, respectively.

In terms of bevacizumab-related adverse events, 8 patients (6.96%) in the BC group experienced hypertension, and 9 patients (7.83%) experienced proteinuria, while no such adverse events were observed in the IC group. The differences between the two groups were statistically significant, with *P* values of 0.022 and 0.012, respectively. These events were considered specific adverse events associated with the bevacizumab regimen. Detailed safety results are shown in **Table 5**.

**Table 5:** Comparison of adverse events between the two patient groups.

Adverse reaction	IC group	BC group	χ^2^	P
Incidence of all adverse events	61 (77.22)	83(72.17)	0.622	0.505
Neutropenia	16 (20.25)	16 (13.91)	1.367	0.242
Leukopenia	11 (13.92)	24 (20.87)	1.528	0.216
Anemia	15 (18.99)	14 (12.17)	1.710	0.191
Thrombocytopenia	3 (3.80)	4 (3.48)	0.014	0.907
Acute liver injury	8 (10.13)	9 (7.83)	0.310	0.578
Acute kidney injury	6 (7.59)	5 (4.35)	0.923	0.337
Hepatitis	4 (5.06)	5 (4.35)	0.054	0.816
Nausea	14 (17.72)	23 (20.00)	0.158	0.691
Vomiting	13 (16.46)	19 (16.52)	0.000	1.000
Loss of appetite	8 (10.13)	14 (12.17)	0.195	0.659
Diarrhea	3 (3.8)	4 (3.48)	0.014	0.907
Rash	7 (8.86)	3 (2.61)	3.744	0.094
Thyroid dysfunction	7 (8.86)	0 (0.00)	10.571	0.002
Immune-related pneumonitis	3 (3.80)	0 (0.00)	4.436	0.066
Immune-related hepatitis	4 (5.06)	0 (0.00)	5.945	0.026
Hypertension	0 (0.00)	8 (6.96)	5.732	0.022
Proteinuria	0 (0.00)	9 (7.83)	6.483	0.012

### 3.4 Overall population subgroup analysis

**Figure 3:**
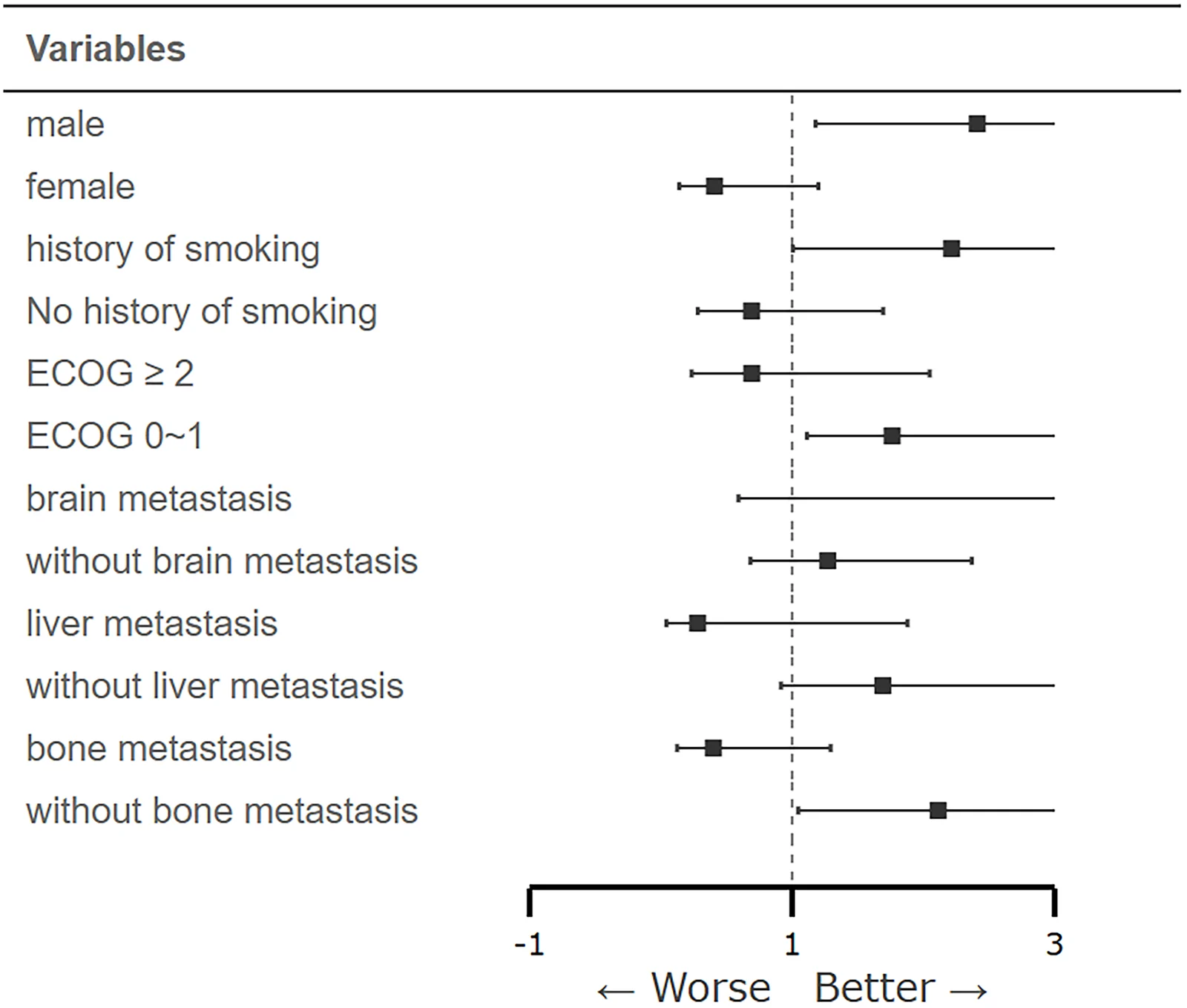
Forest plot of progression-free survival (PFS) subgroup analysis. Subgroup analysis of PFS comparing the risk of disease progression between the ICIs combined with chemotherapy group (IC group) and the bevacizumab combined with chemotherapy group (BC group) within each subgroup (total N = 194). Subgroup factors included gender (male/female), smoking history (yes/no), ECOG performance status (0-1/>=2), and metastatic site status (brain, liver, bone: present/absent). Hazard ratios (HRs) and 95% confidence intervals (CIs) were estimated using Cox proportional hazards regression models. Each subgroup row displays the HR and 95% CI; the size of each square is proportional to the sample size of the corresponding subgroup, and the horizontal line represents the 95% CI range. The vertical dashed line (HR = 1.0) represents the equivalence line: squares to the right of the line (HR>1) indicate a higher risk of disease progression in the BC group (i.e., the IC group is more advantageous); squares to the left of the line (HR<1) indicate a lower risk of disease progression in the BC group (i.e., the BC group is more advantageous). All statistical tests were two-sided with significance level alph α=0.05. HR, hazard ratio; CI, confidence interval; IC group, immune checkpoint inhibitors combined with chemotherapy group; BC group, bevacizumab combined with chemotherapy group; ECOG, Eastern Cooperative Oncology Group; PFS, progression-free survival.

To further identify potential beneficiary groups, this study conducted subgroup analyses of the overall population based on different clinical characteristics. The stratification factors included gender, smoking history, ECOG score, and different metastatic sites. A forest plot was drawn for analysis. Specific results are shown in **Table 6**, and the forest plot is shown in **Figure 3**.

**Table 6:** Subgroup analysis of the overall population.

Subgroup	IC group vs. BC group PFS HR (95%CI)	P
Male	2.410 (1.179 - 4.924)	0.015
Female	0.409 (0.140 - 1.200)	0.100
history of smoking	2.215 (1.007 - 4.876)	0.047
No history of smoking	0.691 (0.282 - 1.693)	0.418
ECOG ≥ 2 point	0.692 (0.234 - 2.048)	0.506
ECOG 0 - 1point	1.762 (1.113 - 3.504)	0.032
With brain metastasis	3.600 (0.591 - 21.932)	0.151
Without brain metastasis	1.272 (0.683 - 2.369)	0.447
With liver metastasis	0.280 (0.042 - 1.878)	0.178
Without liver metastasis	1.691 (0.915 - 3.126)	0.093
With bone metastasis	0.400 (0.124 - 1.293)	0.120
Without bone metastasis	2.112 (1.0461- 4.265)	0.036

Subgroup analysis showed that male patients with smoking history, ECOG 0–1, and without bone metastases had significantly increased risk of disease in the IC group(*P* < 0.05), suggesting bevacizumab-based therapy may be superior in these subgroups.

## 4 Discussion

This study indicates that in patients with advanced non-squamous NSCLC who are negative for driver genes, ICIs combined with chemotherapy demonstrate superior short-term efficacy and PFS benefits compared to bevacizumab combined with chemotherapy. Specifically, the objective response rate in the IC group was significantly higher than that in the BC group, at 41.77% vs 26.09% (*P* = 0.022); furthermore, median PFS in the IC group was also significantly longer than in the BC group, at 14.330 months versus 9.600 months (*P* = 0.014). The above results are generally consistent with previous studies showing that ICIs combined with chemotherapy can provide survival benefits in non-squamous NSCLC (6, 7). In addition to the overall efficacy differences, this study also identified marked differences in the safety profiles between the two groups and revealed heterogeneity across PD-L1 expression levels and some clinical subgroups. These findings collectively form the basis for the subsequent interpretation of results and analysis of clinical implications.

From a mechanistic perspective, the efficacy of ICIs combined with chemotherapy is superior to that of bevacizumab combined with chemotherapy, a superiority potentially attributable to its multistep enhancement of anti-tumor immune responses. The core mechanism of ICIs lies in blocking PD-1/PD-L1-mediated immunosuppressive signals, thereby restoring the anti-tumor activity of T cells and enhancing the immune-mediated clearance of tumor cells (14). Complementarily, chemotherapy not only exerts cytotoxic effects but may also further enhance the effect of immunotherapy by remodeling the tumor immune microenvironment (15). Studies have shown that platinum-based drugs can induce immunogenic cell death, promote the release of tumor-associated antigens, and enhance the activation of antigen-presenting cells, thereby increasing the strength of anti-tumor immune responses (16). In addition, metabolic antagonists such as pemetrexed may upregulate PD-L1 expression on the surface of tumor cells, thereby improving the tumor’s sensitivity to immunotherapy (17). Therefore, there may be a clear synergistic effect between immunotherapy and chemotherapy, which can to some extent explain the advantages of the IC group in ORR and PFS observed in this study.

It should be noted that the ICIs used in this study encompassed multiple agents, including pembrolizumab, atezolizumab, toripalimab, sintilimab, camrelizumab, and tislelizumab. While all these agents share the common mechanism of blocking the PD-1/PD-L1 axis, there are notable differences among them that may influence clinical outcomes. Pembrolizumab and nivolumab are humanized IgG4 anti-PD-1 antibodies, whereas atezolizumab is an engineered IgG1 anti-PD-L1 antibody; the latter may theoretically spare the PD-L2/PD-1 interaction on T cells, potentially reducing certain immune-related adverse events (18). The domestically developed ICIs used in this study—toripalimab, sintilimab, camrelizumab, and tislelizumab—have each demonstrated efficacy in Chinese NSCLC populations through dedicated Phase III trials (e.g., CHOICE-01, ORIENT-11, CAMEL, RATIONALE-304), but they differ in antibody engineering, binding affinity, pharmacokinetic properties, and specific safety profiles. For example, camrelizumab is associated with a unique adverse event of reactive capillary hemangioproliferation (RCCEP), occurring in approximately 30–40% of patients, while tislelizumab was specifically engineered with Fc-silencing to minimize antibody-dependent cellular cytotoxicity (ADCC), potentially reducing off-target toxicity (19). Toripalimab and sintilimab have shown comparable efficacy to pembrolizumab in Chinese populations but with potentially different irAE spectra. In clinical practice, the choice of specific PD-1/PD-L1 inhibitor is typically guided by factors including indication approval status (CSCO/NMPA), reimbursement policies under China’s National Medical Insurance, PD-L1 expression levels (some agents have biomarker-driven indications), comorbidity profile (e.g., avoiding camrelizumab in patients concerned about RCCEP cosmetic effects), prior adverse event history, and drug accessibility and regional availability. However, due to the retrospective nature of this study and the relatively small sample size per agent subgroup, we were unable to perform head-to-head comparisons among individual ICIs. Future studies with larger cohorts, preferably multicenter, are needed to elucidate potential differential efficacy and safety profiles among these agents in the context of combination chemotherapy.

In contrast, bevacizumab’s primary mechanism involves inhibiting the vascular endothelial growth factor pathway to block tumor angiogenesis (20). Despite the vascular normalization theory proposing that bevacizumab may indirectly enhance anti-tumor immunity by improving the microenvironment and promoting T-cell infiltration (21), its immunomodulatory potency remains fundamentally distinct from ICIs’ direct immune suppression blockade. These data suggest that although anti-angiogenic therapy has clear anti-tumor activity, its benefit when combined with chemotherapy in driver-negative advanced non-squamous NSCLC may be inferior to ICI-chemotherapy combinations. This implies divergent mechanistic depths and targets between regimens, ultimately reflecting in clinical efficacy differences.

Importantly, the recognition that immune checkpoint blockade and anti-angiogenic therapy exert complementary anti-tumor effects has spurred the development of bispecific antibodies simultaneously targeting PD-1 and VEGF. Ivonescimab (AK112), a novel tetravalent bispecific antibody, has demonstrated particularly promising results in this regard. In the Phase III HARMONi-2 trial (published in The Lancet, 2025), ivonescimab monotherapy demonstrated superior PFS compared with pembrolizumab monotherapy in patients with PD-L1-positive (TPS ≥1%) locally advanced or metastatic NSCLC (median PFS: 11.1 vs 5.8 months; HR = 0.51, *P* < 0.0001) (22). Additionally, other dual immune-angiogenic strategies are under active investigation, including combinations of anti-angiogenic agents with ICIs (e.g., ramucirumab plus pembrolizumab, as explored in the JVNG trial) and novel bispecific constructs targeting alternative immune-angiogenic axes, which have shown encouraging efficacy signals in early-phase trials (23). These emerging strategies may offer a unified approach that simultaneously addresses tumor immune evasion and angiogenesis, potentially overcoming the limitations of sequential or separate administration. However, the optimal integration of these novel agents into existing treatment algorithms, their safety profiles in broader patient populations, and their cost-effectiveness in real-world practice remain to be elucidated. Future studies directly comparing bispecific antibody-based regimens with current standard ICI-chemotherapy and bevacizumab-chemotherapy combinations will be critical for defining the next generation of first-line therapy for driver gene-negative advanced non-squamous NSCLC.

In terms of safety, this study demonstrates divergent adverse event profiles between the two strategies, though both remain manageable. The IC group showed significantly higher incidences of thyroid dysfunction, pneumonitis, and hepatitis (immune-related) versus the BC group, aligning with established patterns in ICIs-related irAEs (24). This implies that ICI-chemotherapy regimens require vigilant monitoring for immune-related adverse events, particularly thyroid, pulmonary, and hepatic dysfunction. Given their organ-specificity and potential delayed onset, early detection is critical to maintain treatment continuity and mitigate severe toxicity risks.

In contrast, no immune-related adverse events were observed in the BC group, but attention still needs to be paid to adverse events related to anti-angiogenic therapy, including hypertension, bleeding, and proteinuria. These adverse reactions are closely related to the pharmacological mechanism of bevacizumab, and their clinical management focus differs from that of irAEs. Therefore, the differences between the two groups should be understood more as variations in the toxicity spectrum rather than a simple comparison of safety. It is noteworthy that there were no statistically significant differences between the two groups in common adverse reactions such as neutropenia, leukopenia, anemia, nausea, and vomiting, indicating that chemotherapy-related toxicity in the combination regimen was overall similar. Overall, ICIs combined with chemotherapy did not show an unacceptable overall toxicity burden, but specific irAEs still require adequate attention in clinical practice; similarly, bevacizumab combined with chemotherapy also requires vigilance for its unique anti-angiogenic therapy-related adverse events. This indicates that the safety characteristics of the two regimens have different focal points but are generally within a manageable range.

PD-L1 expression level is an important biomarker in the stratification of immunotherapy, based on the theoretical premise that there is a close relationship between the degree of tumor immune evasion and the activation status of the PD-1/PD-L1 axis (25). In this study, patients with high PD-L1 expression who received ICIs combined with chemotherapy showed longer median PFS, which is consistent with findings from studies such as KEYNOTE-024 that conclude ‘patients with high PD-L1 expression are more likely to derive greater benefit from immunotherapy’ (26). This result further supports the clinical value of PD-L1 as a predictive marker for immunotherapy benefits and also suggests that in advanced driver gene-negative non-squamous non-small cell lung cancer, patients with high PD-L1 expression may be the population for whom ICIs combined with chemotherapy is more advantageous.

However, for patients with low or negative PD-L1 expression, this study did not observe a statistically significant difference in PFS between the two groups. This result should be interpreted with caution. On one hand, it suggests that the incremental benefit from ICIs combined with chemotherapy for this subset of patients may be limited; on the other hand, a clear treatment recommendation cannot be made based solely on this finding. At present, a more reasonable interpretation is that bevacizumab combined with chemotherapy may still be considered as one of the alternative options for populations with low or negative PD-L1 expression. Meanwhile, this result also indicates that a single PD-L1 indicator may be insufficient to fully identify populations that may benefit from immunotherapy, and further studies incorporating additional biomarkers are needed in the future, such as tumor mutation burden (27), microsatellite instability (28), and gene expression profiles (29), to improve the accuracy of patient selection. However, these indicators were not systematically included in the analysis of this study, so their clinical significance still needs to be validated with more robust evidence.

In the subgroup analysis, this study found that in males, patients with a history of smoking, those with an ECOG score of 0–1, and those without bone metastases, the risk of disease progression in the IC group was significantly increased. This result should not be directly interpreted as a negation of the overall main analysis conclusion, but rather should be seen as an exploratory finding indicating potential heterogeneity. Theoretically, patients with different clinical characteristics may have differences in tumor biological behavior, tumor microenvironment status, and treatment sensitivity, which may lead to the relative benefit of different combination regimens not being entirely consistent in certain populations. In the context of this study, these subgroup results suggest that bevacizumab combined with chemotherapy may still have relative advantages in certain specific patients, and the related mechanisms may be associated with anti-angiogenic effects and the modulation of the tumor microenvironment.

It should be emphasized that subgroup analyses are inherently exploratory in nature, and their conclusions are easily affected by factors such as insufficient sample size, imbalance between groups after stratification, and residual confounding. Therefore, differences observed in subgroups such as males, smokers, patients with ECOG scores of 0–1, and those without bone metastases should be interpreted with caution. In other words, these results are more suitable as a source of hypotheses for subsequent research and should not be directly extrapolated as a definitive basis for clinical decision-making. In clinical practice, treatment choices still need to be made based on a comprehensive assessment of the patient’s overall baseline characteristics, disease burden, tolerability, and potential benefits, rather than simply drawing conclusions based on a single subgroup characteristic.

This study still has several limitations. First, this study’s sample size was limited, which may affect statistical power and limit the stability and interpretability of some subgroup analysis results; consequently, further validation of the conclusions of this study is still needed in larger samples. Second, this study currently lacks longer-term follow-up and thus is unable to adequately assess overall survival outcomes. For advanced non-squamous NSCLC, overall survival remains an important endpoint for evaluating the long-term value of treatment, so extending follow-up in the future is necessary.

In addition, this study is a single-center retrospective study, and its inherent limitations include potential selection bias, information bias, and the influence of confounding factors. these limitations may introduce confounding in efficacy assessment and measurement errors in safety evaluation and limit the external generalizability of the results. Although this study attempted to minimize the impact of bias through strict inclusion and exclusion criteria and statistical analysis, the retrospective design itself still makes it difficult to completely avoid residual confounding. Therefore, the results of this study are more suitable to serve as real-world evidence and provide a reference for subsequent research, while their external validity still needs to be further confirmed by multicenter, prospective randomized controlled trials. Overall, the results of this study suggest that in patients with advanced non-squamous non-small cell lung cancer without driver gene mutations, ICIs combined with chemotherapy may have better efficacy compared to bevacizumab combined with chemotherapy, but the relevant subgroup differences and long-term survival value still need to be further clarified in higher-quality studies.

## 5 Conclusion

The results of this study indicate that in the first-line treatment of advanced non-squamous NSCLC patients without driver gene mutations, ICIs combined with chemotherapy show superior efficacy in both objective response rate and progression-free survival compared with bevacizumab combined with chemotherapy.

In terms of safety, ICIs combined with chemotherapy are generally well tolerated, and the related adverse reactions are within a manageable range, suggesting that this treatment regimen has overall manageable safety.

In addition, this study observed that patients with high PD-L1 expression benefited more significantly after receiving ICIs combined with chemotherapy. The above findings suggest that ICIs combined with chemotherapy can provide a reference for clinical first-line treatment options and individualized treatment decisions for such patients.

## Data Availability

The raw data supporting the conclusions of this article will be made available by the authors, without undue reservation.
